# Design of Driving Waveform for Shortening Red Particles Response Time in Three-Color Electrophoretic Displays

**DOI:** 10.3390/mi12050578

**Published:** 2021-05-19

**Authors:** Wenjun Zeng, Zichuan Yi, Xichen Zhou, Yiming Zhao, Haoqiang Feng, Jianjun Yang, Liming Liu, Feng Chi, Chongfu Zhang, Guofu Zhou

**Affiliations:** 1 College of Electron and Information, University of Electronic Science and Technology of China, Zhongshan Institute, Zhongshan 528402, China; zwjcareer@163.com (W.Z.); zxc_2021_4_15@163.com (X.Z.); ym1679@sina.com (Y.Z.); haoqiang.feng@m.scnu.edu.cn (H.F.); sdyman@uestc.edu.cn (J.Y.); liulmxps@126.com (L.L.); chifeng@semi.ac.cn (F.C.); cfzhang@uestc.edu.cn (C.Z.); 2 South China Academy of Advanced Optoelectronics, South China Normal University, Guangzhou 510006, China; guofu.zhou@m.scnu.edu.cn

**Keywords:** electrophoretic displays, driving waveform, response time, reference gray scale, red particles

## Abstract

Three-color electrophoretic displays (EPDs) have the advantages of multi-color display and low power consumption. However, their red particles have the disadvantage of long response time. In this paper, a driving waveform, which is based on electrophoresis theory and reference gray scale optimization, was proposed to shorten the response time of red particles in three-color EPDs. The driving waveform was composed of erasing stage, reference gray scale forming stage, red driving stage, and white or black driving stage. Firstly, the characteristics of particle motion were analyzed by electrophoresis theory and Stokes law. Secondly, the reference gray scale of the driving waveform was optimized to shorten the distance between red particles and a common electrode plate. Finally, an experimental platform was developed to test the performance of the driving waveform. Experimental results showed that the proposed driving waveform can shorten the response time of red particles by 65.57% and reduce the number of flickers by 66.67% compared with the traditional driving waveform.

## 1. Introduction

Three-color electrophoretic displays (EPDs) are a new type of electronic paper [1,2,3,4]. They effectively make up for the limitation of traditional EPDs in the performance of multi-color display [5,6,7]. However, the driving time of red particles is much longer than that of black particles and white particles, which affects the visual effect of three-color EPDs [4,8]. Therefore, certain improvements are required for commercialization of three-color EPDs, such as shortening the response time of red particles. Driving waveforms are a voltage sequence applied to EPDs, which can control the motion of particles [9,10,11]. Traditional driving waveforms of three-color EPDs have the problem of a long period, which prolongs the response time of red particles [12]. Therefore, it is of great significance to shorten the response time of red particles in three-color EPDs by optimizing the driving waveform.

The response time of particles is mainly determined by its motion [13], and the motion of particles can be controlled by the electric field according to the driving waveform [14]. Additionally, the electric field physical model has been proposed [15], which laid a theoretical foundation for the optimization of driving waveforms. The activity of particles was determined by the activation stage of driving waveforms, and it has a great impact on the response speed of particles [16]. A driving waveform for optimizing the activation stage was proposed [17], an inflection point was used as the duration for improving the activity of particles, and it can effectively shorten the driving time of the activation stage. However, this method did not follow the direct current (DC) balance, and EPDs would be damaged easily [18]. The response time was related to the distance between particles and the common electrode plate of EPDs, and this distance was determined by the reference gray scale of EPDs. A driving waveform based on optimizing gray scale conversion paths was proposed to shorten the response time [19]. However, the residence time of the particles has a great influence on the response speed of particles [20]. The longer the residence time of particles, the slower the response speed. Furthermore, new materials for EPDs are also beneficial to improve the response speed of particles [21,22], porous silica nanoparticles and silica coated with ionic liquid polymer nanoparticles were proposed for shortening the response time in EPDs [23]. The design of driving algorithms has greatly improved the display quality [24]. A driving algorithm based on a convolutional neural network was proposed to improve the display quality in EPDs [25], which provided a new method for optimizing driving waveforms.

In this paper, a new driving waveform was proposed for shortening the response time of red particles in three-color EPDs. Electrophoresis theory and Stokes law were used to analyze the motion characteristics of particles. Then, the distance between red particles and a common electrode plate was shortened by optimizing reference gray scales, which can shorten the response time of red particles.

## 2. Principle

### 2.1. Principle of Three-Color EPDs

**Figure 1:**
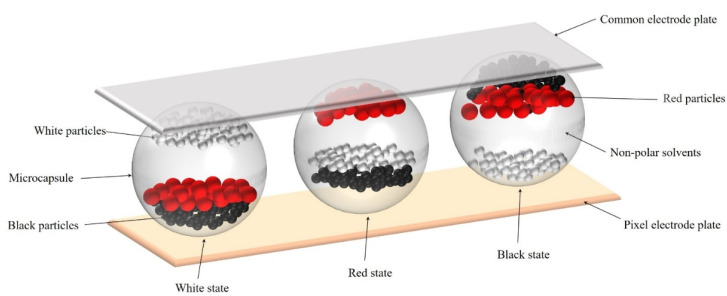
The structure of a three-color EPD. It is mainly composed of a common electrode plate, a pixel electrode plate, black particles, white particles, red particles, and non-polar solvents. The white state is the situation when white particles are driven to the common electrode plate. The red state is the situation when red particles are driven to the common electrode plate. The black state is the situation when black particles are driven to the common electrode plate.

The three-color EPD is based on microcapsule technology. Its structure is shown in Figure 1. It is mainly composed of a common electrode plate, a pixel electrode plate, black particles, white particles, red particles, and non-polar solvents [26]. These three kinds of particles have different kinds of charges, and they are encapsulated in microcapsules. The common electrode plate and the pixel electrode plate are composed of transparent glass substrates covered with indium tin oxide (ITO). The microcapsule is sandwiched between the common electrode plate and the pixel electrode plate. White particles with negative charge are driven to the common electrode plate when a negative voltage is applied to the pixel electrode plate, and at this time, the three-color EPD is in white state. On the contrary, black particles and red particles with positive charges are driven to the common electrode plate when a positive voltage is applied to the pixel electrode plate. However, the amount of charge of red particles is different from that of black particles. Therefore, there are different situations according to the amplitude of the applied positive voltage. The threshold voltage of red particles is lower than that of black particles, so red particles are driven to the common electrode plate when a low positive voltage is applied. At this time, the three-color is in red state. In addition, black particles are driven to the common electrode plate when a high positive voltage is applied, and at this time, the three-color EPD is in black state. The three-color EPD would be maintained at the initial state when no voltage is applied to the pixel electrode plate, which can reduce the energy consumption [27,28].

### 2.2. Motion Analysis of Particles

The analysis of particle motion plays an important role in shortening the response time of the three-color EPD. A particle in a microcapsule is mainly affected by gravity, buoyancy, electrostatic force, and viscosity of the non-polar solvent [29]. The density of the non-polar solvent is equal to that of the particle. Otherwise, the phenomenon of particle sedimentation would occur. The relationship between the gravity and the buoyancy of the particle is shown in Equation (1).

(1) $$\frac{4}{3}\pi R^{3}\left(\rho_{l}-\rho_{m}\right)=0\;$$

where R is the radius of the particle, $\rho_{l}$ is the density of the non-polar solvent, $\rho_{m}$ is the density of the particle. The relationship between the velocity and the resultant force of the particle can be obtained by Newton second law [30], and it is shown in Equation (2).

(2) $$\frac{Uq}{d}-6\pi \eta \nu R=m\frac{d\nu }{dt}\;$$

where U is the voltage applied to the pixel electrode plate, q is the amount of charge carried by the particle, d is the distance between the common electrode plate and the pixel electrode plate, η is the viscosity coefficient of the non-polar solvent, ν is the velocity of the particle. In Equation (2), the first term is the electrostatic force. The second term is the viscous of the non-polar solvent, and it is calculated by Stokes law.

(3) $$\nu =\frac{Uq}{6\pi d\eta R}\left(1\pm e^{-\frac{6\pi \eta R}{m}t}\right)$$

The velocity of the particle can be obtained by solving Equation (2), and it is shown in Equation (3). It can be seen that the velocity of the particle is proportional to the applied voltage. The higher the applied voltage, the faster the velocity of the particle. However, red particles and black particles have the same charge polarity, and black particles move faster than red particles when a high voltage is applied to the three-color EPDs. Therefore, the response time of red particles cannot be shortened by increasing the amplitude of the applied voltage. The response time of red particles can be obtained by Equation (4).

(4) $$T_{r}=\frac{6\pi sd\eta R}{Uq\left(1\pm e^{-\frac{6\pi \eta R}{m}t}\right)}$$

where s is the distance between red particles and the common electrode plate. It can be seen that the response time of red particles is proportional to s. So, the response time of red particles can be shortened by reducing the distance between red particles and the common electrode plate. s is related to the reference gray scale of three-color EPDs. s is increased when the reference gray scale is white gray scale, while s is decreased when the reference gray scale is black gray scale.

## 3. Experiment and Discussion

### 3.1. Experimental Platform

**Figure 2:**
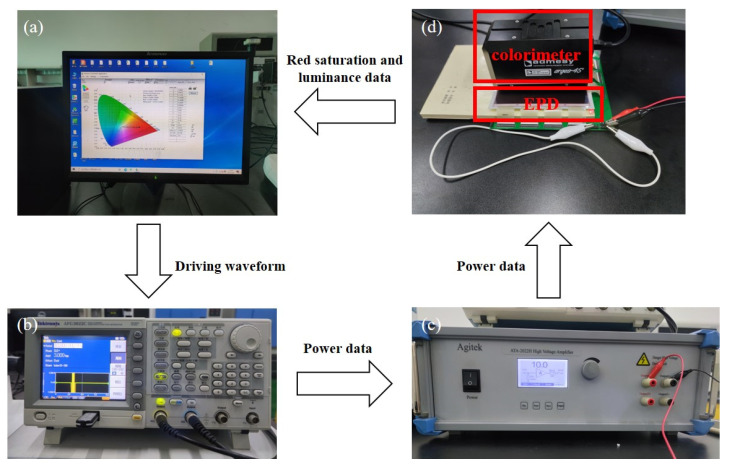
The experimental platform for three-color EPDs. (**a**) A computer. (**b**) A function generator. (**c**) A voltage amplifier. (**d**) A colorimeter and a three-color EPD. The driving waveform was edited in the computer. Then, an edited driving waveform was imported into the function generator, and then power data from the function generator was amplified by the voltage amplifier. Next, the three-color EPD was driven by power from the voltage amplifier, red saturation and luminance data of the three-color EPD were collected by the colorimeter. Finally, the data were transmitted to the computer.

The performance of a three-color EPD can be determined by its red saturation, luminance, and response time. The state of red particles can be expressed by the red saturation, and the state of black particles and white particles can be expressed by the luminance. Therefore, an experimental platform was developed to test these parameters. The experimental platform is shown in Figure 2. It was composed of a driving system and a testing system. The driving system was composed of a computer (H430, Lenovo, Beijing, China), a function generator (AFG3022C, Tektronix, Beaverton, OR, USA), and a voltage amplifier (ATA-2022H, Agitek, Xian, China), which was used to generate driving waveforms. The testing system was composed of the computer and a colorimeter (Arges-45, Admesy, Ittervoort, The Netherlands), which was used to record the red saturation and the luminance data of three-color EPDs.

In this experiment, a three-color EPD was used as the tested object. In the testing process, the driving waveform was edited by Arbexpress (V3.4, Tektronix, Beaverton, OR, USA) waveform editing software in the computer. Then, an edited driving waveform could be imported into the function generator by a universal serial bus (USB) interface, and then it was amplified by the voltage amplifier. Next, the three-color EPD was driven by power from the voltage amplifier, red saturation and luminance data of the three-color EPD were collected by the colorimeter. Finally, the data were transmitted to the computer, and Admesy software was used to record these data in real time.

### 3.2. Design of Driving Waveform

**Figure 3:**
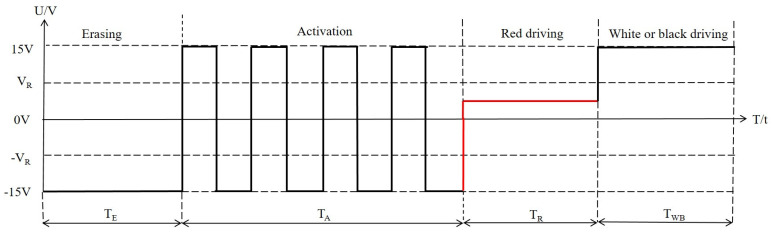
The traditional driving waveform of three-color EPDs. It was composed of an erasing stage, an activation stage, a red driving stage, and a white or a black driving stage. $T_{E}$ is the driving time of the erasing stage, $T_{A}$ is the driving time of the activation stage, $T_{R}$ is the driving time of the red driving stage, $T_{WB}$ is the driving time of the white or the black driving stage. $V_{R}$ is the driving voltage of red particles.

The traditional driving waveform of three-color EPDs was composed of an erasing stage, an activation stage, a red driving stage, and a white or a black driving stage, as shown in Figure 3 [12]. The driving waveform was followed the DC balance. In the erasing stage, a negative driving voltage was applied to erase original images. In the activation stage, four cycles of square wave pulse were applied to reduce viscous force. Then, the activity of particles could be increased, and a white reference gray scale was formed. The red driving stage was a low positive DC voltage $V_{R}$ to drive red particles. The white or black driving stage was a +15 V DC voltage. However, the reference gray scale formed by the tradition driving waveform was a white state, which could increase the distance between red particles and the common electrode, and the response time of red particles was prolonged. In addition, the driving time of the activation stage and erasing stage were prolonged by the DC balance, which prolonged the driving time of red particles.

**Figure 4:**
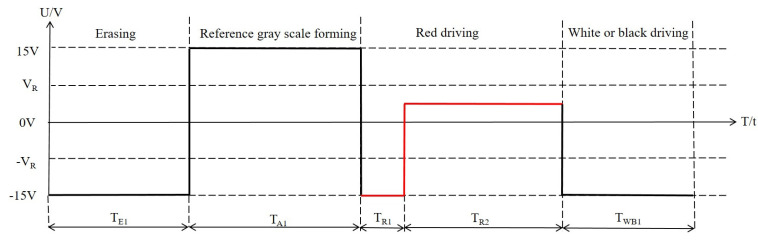
The proposed driving waveform of three-color EPDs. It was composed of an erasing stage, a reference gray scale forming stage, a red driving stage, and a white or a black driving stage. $T_{E1}$ is the driving time of the erasing stage, $T_{A1}$ is the driving time of the activation stage, $T_{R1}$ is the driving time of a −15 V driving voltage in the red driving stage, $T_{R2}$ is the driving time of a positive DC voltage in the red driving stage, $T_{WB1}$ is the driving time of the white or the black driving stage. $V_{R}$ is the driving voltage of red particles.

We proposed a driving waveform to shorten the response time of red particles in three-color EPDs. It was composed of an erasing stage, a reference gray scale forming stage, a red driving stage, and a white or a black driving stage, as shown in Figure 4. In the erasing stage, a negative driving voltage was applied to erase original images. Then, a positive driving voltage was applied to form a black reference gray scale in the reference gray forming stage. In the red driving stage, a short −15 V driving voltage was applied to optimize the reference gray scale, and then a low positive DC voltage $V_{R}$ was applied to drive red particles. The white or black driving stage was a −15 V DC voltage. The driving time of each driving state was followed by Equation (5) to reduce residual charges.

(5) $$T_{A1}=T_{E1}+T_{R1}+T_{WB1}\;$$

where $T_{A1}$ is the driving time of the activation stage, $T_{E1}$ is the driving time of the erasing stage, $T_{R1}$ is the driving time of a −15 V driving voltage in the red driving stage, $T_{WB1}$ is the driving time of the white or the black driving stage.

### 3.3. Optimal Driving Voltage for Red Particles

**Figure 5:**
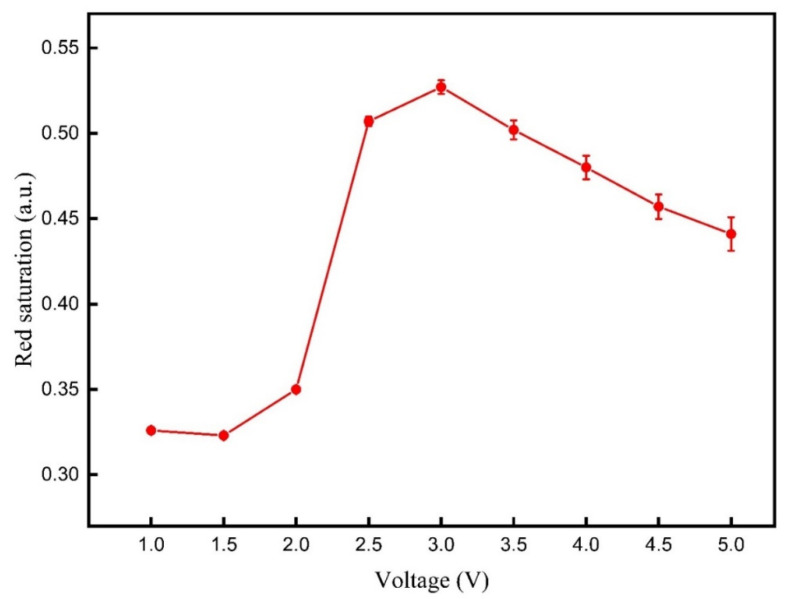
The red saturation of the three-color EPD when it was driven by different DC voltages. The red saturation increased with the increase of the DC voltage from 1 to 3 V. On the contrary, the red saturation decreased with the increase of the DC voltage from 3 to 5 V. The maximum red saturation was 0.527 when the DC voltage was 3 V.

The three-color EPD was driven by different DC voltages to determine the optimal driving voltage for red particles. The red saturation values of the three-color EPD driven by different DC voltages are shown in Figure 5. It can be seen that the red saturation was increased with the increase of the DC voltage from 1 to 3 V. At this time, the applied voltage was not high enough to drive black particles, and red particles can be driven to the common electrode plate. On the contrary, the red saturation was decreased with the increase of the DC voltage from 3 to 5 V. The reason for this phenomenon was that some black particles were mixed with red particles due to an increase of electrostatic force on black particles. The maximum red saturation value was 0.527 when the DC voltage was 3 V. Therefore, the driving voltage of red particles $V_{R}$ was set to 3 V in the proposed driving waveform.

### 3.4. Optimization of the Reference Gray Scale

**Figure 6:**
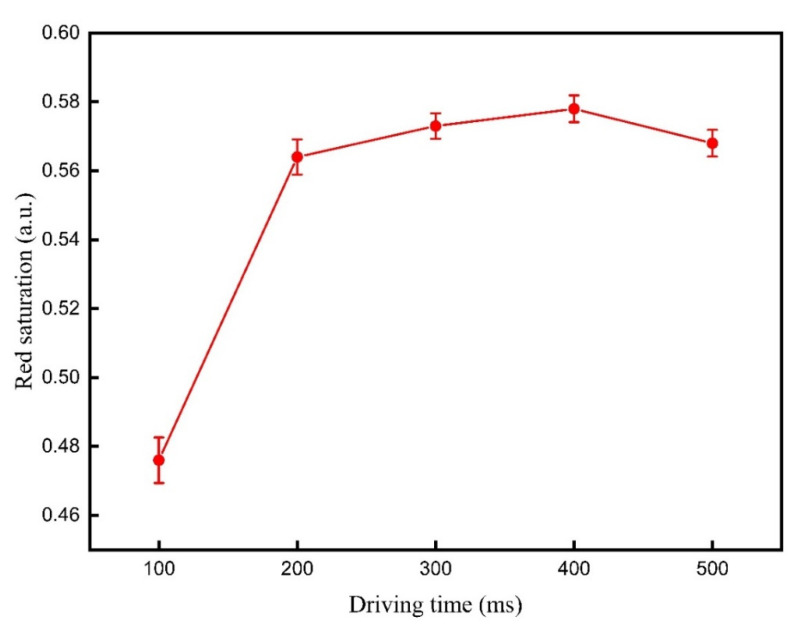
The red saturation of the three-color EPD when it was driven by different driving times of $T_{A1}$. It was increased with the increase of $T_{A1}$ from 100 to 400 ms, and the red saturation was decreased when $T_{A1}$ reached 500 ms.

It is necessary to determine an optimal reference gray scale to get an optimal motion distance of red particles. Firstly, the reference gray scale forming stage of the proposed driving waveform has an activating function on particles, so the driving time of this stage must be determined. In the driving waveform, $T_{R2}$ was set to 5000 ms, $T_{R1}$ was equal to $T_{A1}$. Then, $T_{A1}$ was set to 100, 200, 300, 400, and 500 ms. Red saturation values driven by different $T_{A1}$ values are shown in Figure 6. It can be seen that the red saturation was increased with the increase of $T_{A1}$ from 100 to 400 ms, and the red saturation was decreased when $T_{A1}$ reached 500 ms. This phenomenon indicated that red particles were obtained from the optimal activity when $T_{A1}$ was 400 ms. At this time, the viscosity from non-polar solvents on red particles was the minimum. Therefore, the driving time of the reference gray scale forming stage was set to 400 ms in the proposed driving waveform.

**Figure 7:**
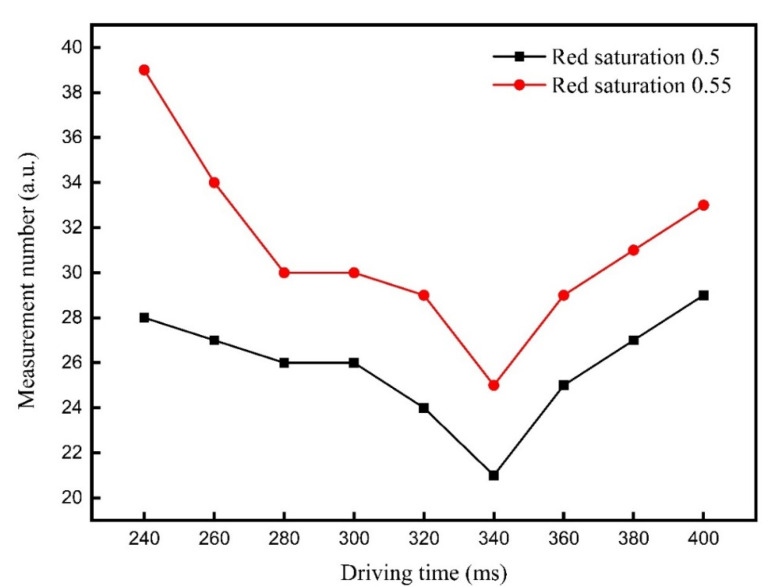
The response time of red saturation 0.5 and red saturation 0.55 driven by different $T_{R1}$ values are shown in Figure 7. The response time of red saturation 0.55 and red saturation 0.5 decreased with the increase of $T_{R1}$ from 240 to 340 ms. Then, the response time of red saturation 0.55 and red saturation 0.5 increased with the increase of $T_{R1}$ from 340 to 400 ms.

The three-color EPD can form a black reference gray scale in the reference gray scale forming stage. However, the reference gray scale needed to be optimized to separate black particles, otherwise the three-color EPD cannot get a high saturation red. The method to form a new reference gray scale was to control the driving time of $T_{R1}$, which can control the position of black and white particles. $T_{R1}$ was set to 220–400 ms for testing the response time of red particles. The response time of red saturation 0.5 and red saturation 0.55 driven by different $T_{R1}$ values are shown in Figure 7. Measurement number was the number of measurements tested by the colorimeter, and the measurement interval was 0.11 s. It can be seen that the response time of red saturation 0.55 was the longest when $T_{R1}$ was 240 ms. The reason for this phenomenon was that the driving time $T_{R1}$ was not long enough to separate black particles. Some black particles were mixed with red particles, so it was difficult for the three-color EPD to achieve a high red saturation. The response time of red saturation 0.55 and red saturation 0.5 were decreased with the increase of $T_{R1}$ from 240 to 340 ms, and they can achieve the shortest response time at 340 ms. In this time, red particles and black particles were separated, so a high red saturation can be achieved. The response time of red saturation 0.55 and red saturation 0.5 were increased with the increase of $T_{R1}$ from 340 to 400 ms. The reason for this phenomenon was that the distance between red particles and the common electrode plate was increased with the increase of $T_{R1}$. Meanwhile, white particles would be pulled to the common electrode plate with the application of negative voltage, which could increase the response time of red particles. In summary, the shortest response time can be obtained when $T_{R1}$ was 340 ms. At this time, the response time of red saturation 0.5 was 2.31 s, and the response time of red saturation 0.55 was 2.75 s.

### 3.5. Performance Comparison of Driving Waveforms

**Figure 8:**
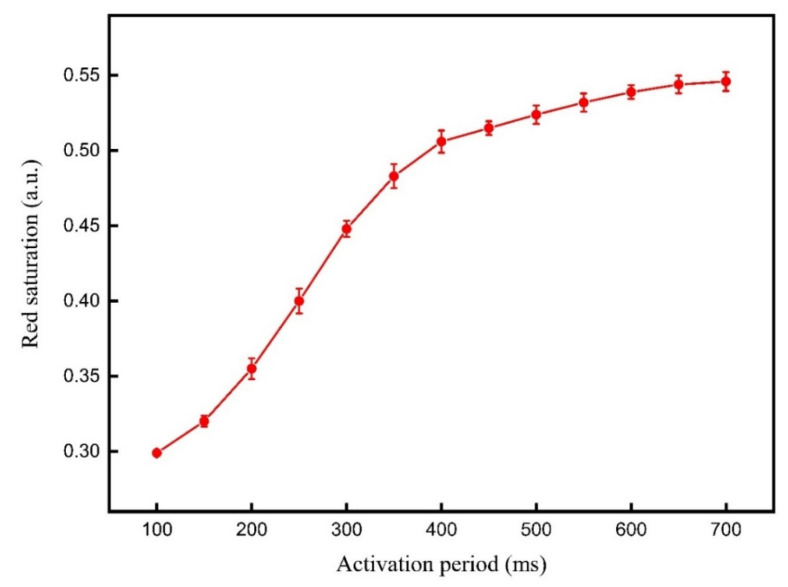
The red saturation achieved by different activation stages of the traditional driving waveform. The red saturation increased with the increase of the activation period, and then it tended to saturation when the activation period reached 650 ms.

The traditional driving waveform [12] was used to compare with the proposed driving waveform. The red driving stage of the traditional driving waveform was set to 5000 ms, which was the same as the proposed driving waveform. Then, the activation stage of the traditional driving waveform was set to 200–700 ms for testing. The red saturation achieved by different activation stages of the traditional driving waveform are shown in Figure 8. It can be seen that the red saturation increased with the increase of the activation period, and then it tended to saturation when the activation period reached 650 ms. It can be concluded that the activity of red particles would be increased with the increase of the activation period in a certain range. However, the period of the driving waveform increased by increasing the activation period, which increased the response time of red particles. Therefore, the activation period of the traditional driving waveform was set to 650 ms due to these two factors. The red saturation of three-color EPDs was 0.544 when the activation period was 650 ms.

**Figure 9:**
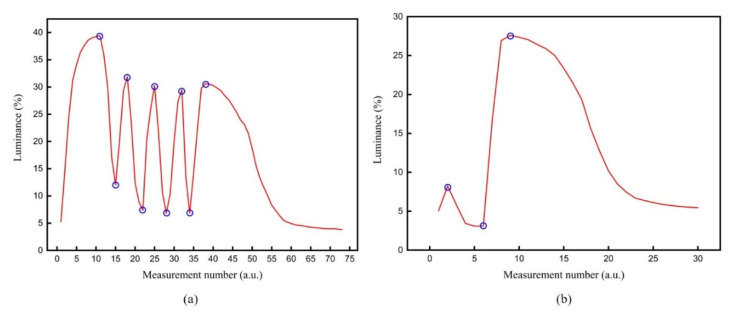
The comparison of flickers between the traditional driving waveform and the proposed driving waveform. (**a**) The luminance curve of the traditional driving waveform. It alternated between bright and dark multiple times. The number of flickers was 9 times, and the maximum intensity was 27.39. (**b**) The luminance curve of the proposed driving waveform. It had fewer peaks, the number of flickers was 3 times, and the maximum intensity of flickers was 24.51.

The flicker of the traditional driving waveform was compared with the flicker of the proposed driving waveform. The luminance curve of the traditional driving waveform is shown in Figure 9a. It can be seen that the luminance curve alternated between bright and dark multiple times because the traditional driving waveform had multiple positive and negative voltage alternating processes. The number of flickers was 9 times, and the maximum intensity was 27.39. The luminance curve of the proposed driving waveform is shown in Figure 9b. It can be seen that there were fewer peaks, the number of flickers was 3 times, and the maximum intensity of flickers was 24.51. Therefore, the proposed driving waveform can reduce the number of flickers by 66.67% and reduce the flicker intensity by 10.51 compared with the traditional driving waveform.

**Figure 10:**
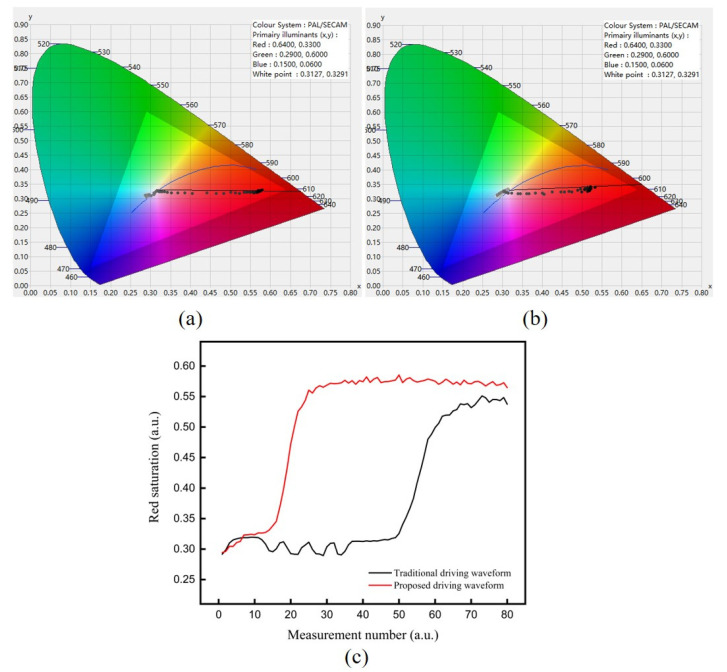
The red saturation when the three-color EPD was driven by the proposed driving waveform and the tradition driving waveform. (**a**) The red saturation of the three-color EPD achieved by the tradition driving waveform. (**b**) The red saturation of the three-color EPD achieved by the proposed driving waveform. (**c**) The comparison of the response time between the proposed driving waveform and the tradition driving waveform.

The response time of red particles driven by the proposed driving waveform was compared with that of the traditional driving waveform. The red saturation of the three-color EPD achieved by the proposed driving waveform is shown in Figure 10a. The red saturation of the three-color EPD achieved by the tradition driving waveform is shown in Figure 10b. The comparison of the response time driven by the proposed driving waveform and the tradition driving waveform is shown in Figure 10c. The black line represented the traditional driving waveform, and the red line represented the proposed driving waveform. It can be seen that the red saturation of the black line fluctuated around 0.31 because of a long period of the activation stage, which prolonged the response time of particles. The response time of red saturation 0.5 driven by the traditional driving waveform was 6.71 s. On the contrary, the red saturation of the red line could rise rapidly. The response time of red saturation 0.5 driven by the proposed driving waveform was 2.31 s. This phenomenon showed that the proposed driving waveform can effectively shorten the response time of red particles because the distance between red particles and the common electrode plate was shortened, and the period of the driving waveform was decreased. The response time of red particles driven by the proposed driving waveform was shortened by 65.57% compared with the traditional driving waveform.

## 4. Conclusions

In this paper, a driving waveform was proposed to shorten the response time of red particles in three-color EPDs. The driving time and flicker of the driving waveform were effectively reduced by optimizing the activation stage and the erasing stage. In addition, the distance between red particles and the common electrode plate was shortened by optimizing the reference gray scale. Finally, an experimental platform was developed to verify the effectiveness of the driving waveform. It was verified that the driving waveform could effectively shorten the response time of red particles, which provided a certain reference value for the application and development of three-color EPDs.

## Data Availability

Data is contained within the article.
